# Obituary Dr Theo Elsinghorst

**DOI:** 10.1080/01652176.2018.1440720

**Published:** 2018-02-27

**Authors:** Han van der Kolk

**Affiliations:** Swiss Institute for Equine Medicine (ISME), Vetsuisse Faculty, University of Bern, Bern, Switzerland

Veterinary pathologist and publisher who worked on a broad spectrum of animal diseases. Born on 18 May 1936, in Winterswijk, the Netherlands, he died on 7 May 2017, in Bilthoven, the Netherlands, aged 80 years.

**Figure:**
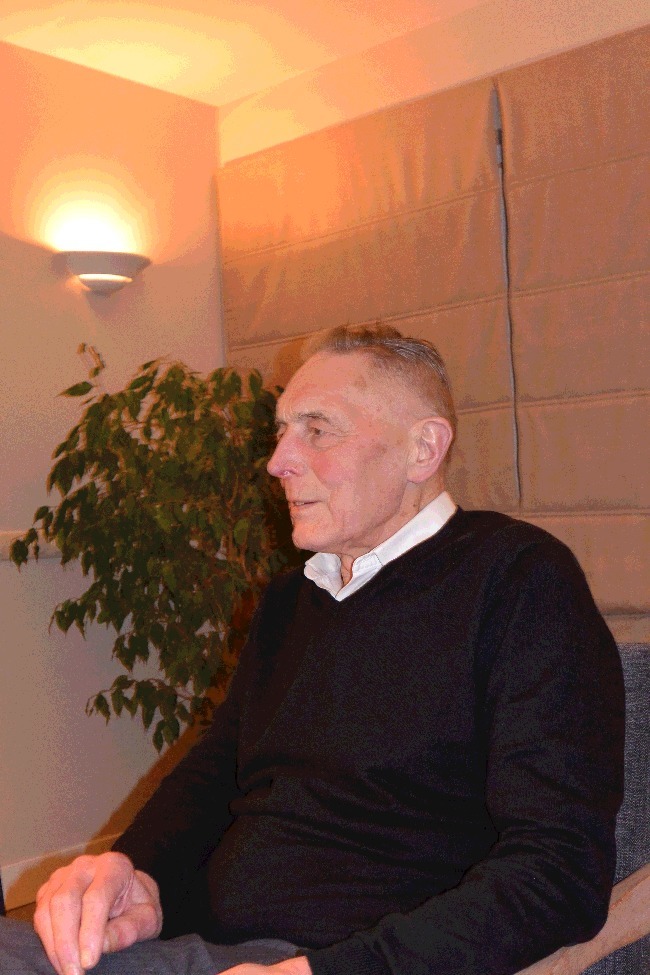

He graduated in 1964 from the Faculty of Veterinary Medicine at the State University of Utrecht, the Netherlands. Since his graduation he had been working there as a member of the staff of the Department of Veterinary Pathology. He successfully defended his PhD thesis on ‘The endometrial carcinoma in the rabbit’ in 1987 and subsequently became a diplomate in pathology of the Royal Netherlands Veterinary Association.

Dr Th.A.M. Elsinghorst was also very interested in books and by founding the company *Euroscience* he became a publisher too. He was very active in that field and one of his achievements was the publication of a veterinary dictionary named ‘Veterinair Ziektekundig Woordenboek’. Furthermore he compiled a database on (bio)medical dissertations.

Previously, the Royal Netherlands Veterinary Association established the *Veterinary Quarterly*, replacing quarterly English issues which used to appear as consecutive numbers of the journal *Tijdschrift voor Diergeneeskunde* in Dutch. From 2002 onwards, the Royal Netherlands Veterinary Association passed over the ownership of the *Veterinary Quarterly* to *Euroscience* with drs Theo Elsinghorst and Jan Vos serving as editors. As a consequence, dr Elsinghorst merely saved the *Veterinary Quarterly* as a journal. Even more he made it successful by his hard work and this success of the journal was reflected by a high impact factor. Drs Theo Elsinghorst and Jan Vos stepped down in 2011 as the journal's editors and Taylor & Francis took over as publisher from *Euroscience*.

Perhaps even more impressive were his bicycle journeys. For example, in spring 2017 he made plans on a bicycle tour via the ACA bicycle route 66 from Chicago to Santa Monica, LA, USA. He used to cycle on the average 125 km per day (or in his words ‘about 4000 km equivalent to 32 days’).

Although Dr Elsinghorst stepped down in 2011 as the journal's editor he always kept in touch for true interest and support thereby reflecting his helpful nature. It was a pleasure and honour working with him.

